# Monthly Summary

**Published:** 1892-05

**Authors:** 


### Monthly Summary.

MELTED GOLD FILLINGS.—For example, we will take a patient for whom three large gold fillings are to be inserted; it will take at least three sittings, often more. Prepare the cavities only at the first sitting, take an impression of them (wax is as good as the other materials), place your temporary filling of wax or gutta-percha, and dismiss your patient. The difference is, you intend to make a perfect filling for these three teeth in your laboratory at your leisure, independent of saliva. Mix enough plaster with fine plumbago to hold the plumbago together, and set; oil the wax impression as for an ordinary plaster cast. While you are preparing the gold, place your mold to dry on the oil stove; use 22, fine gold. The mold can be made of the plaster and plumbago alone, but small flasks in the form of the ordinary molding is better. The plaster molds should be tied together with a narrow band of tin, or stove-pipe iron. Your three molds ready, and placed in convenient position, melt your gold in a small crucible, and pour quickly; it will not do as well to have two fillings in one mold, as one or the other is likely to be imperfect. If the mold is well made, the filling requires but little trimming. You now have three fillings ready. When the patient makes her appearance, you feel no dread of saliva; the first filling is freed from the tooth, the cavity cleansed of wax particles; you try the fit of your gold filling, and trim so it will enter the cavity readily. Test the articulation, and trim accordingly. Everything being ready, a single napkin is all that will be needed. Mix your oxyphosphate thin, that it may adhere to the walls of the cavity and the metal. Line the cavity with it, using but little, though enough to be pressed out. When you press in your solid gold filling, there will remain just enough to fill the imperfect adaptation of the gold, and this is what is most needed. All the fine pits and lines of the cavity are filled. Owing to the nice fit of the gold, there is but little cement exposed around the walls of the cavity to be acted on by secretions, and the action is not the same, owing to the presence of gold, which plays with zinc. It will endure as much as the tooth substance, unless too much is exposed, owing to a bad fit; in such a case, remove a little of the cement, and fill with foil, which is the work of but a few moments. In finishing the filling, do so from the center of the filling towards the surrounding walls, as it spreads the gold over the thin line of the cement. The antagonizing of the teeth will also spread the gold. Still there will be little danger of decomposition, as it is but a film, and preserves bone very well. Keep the tooth dry till the cement is well hardened. Whatever defect occurs to a filling of this kind is from the exterior. Three gold fillings are inserted at one setting, with but little trouble to your patient, and the satisfaction to the dentist that he has inserted solid gold filling that will stand the test of time. In incisors, bicuspids, or any of the front teeth where the cavity is arc-shaped and cutting edge is required, it will be safer to drill a small hole through that part of the filling where, when it comes in contact with the cavity of the tooth, a shallow hole can be drilled into the tooth of two or three threads that will not endanger it, or give the patient much pain; then tap the end of a gold wire the size of the hole in the filling, and screw it in gently. After the cement has hardened, cut it off and finish. Such a filling will stay, and stand to cut in molars. Where it would be deemed necessary, two screws can be inserted. Tin is as good as gold in some cases; it can be cast in the same manner, with little trouble, as it melts easily. The cavity of the tooth should be shaped so as to allow the impression to come out without drag on the wax. Time will be saved by this in the adjustment of the filling. Now we can place a solid gold filling in any tooth, in any part of the mouth, with less trouble, and a more permanent operation, than a small foil filling in the front teeth.—OLIVER MARTIN, in *Dominion Journal.*

---

ENGLISH ADVERTISING DENTISTS.—A law case, bearing on the subject of advertising as used by professional men, was practically closed in the Court of Appeal in London yesterday. A dentist named Partridge, holding a diploma from the Royal College of Surgeons, Dublin, registered by the General Medical Council, set up a dental institution, supported by extensive advertising. The dublin people thereupon canceled his diploma, and the Medical Council withdrew the registration, whereupon Mr. Partridge brought an action, which, though it did not get him back his diploma, had the effect of getting his name restored to the register. Now, this is a very vital part of the matter, because although the diploma itself being canceled, he could not call himself by the titles it conferred, he was still now again a registered dentist, and could recover his charges at law, if need be. All this took a long time, and, unfortunately for Mr. Partridge, the authorities with whom he was in conflict had another string to their bow. For one of the conditions attached to his diploma had been a promise not to advertise, and he had, in point of fact, spent upwards of $50,000 in advertising. The Medical Council, taking advantage of this, again removed the name from the register, on the ground that Mr. Partridge's broken promise constituted "infamous conduct in a professional regard," and his action at law, by way of meeting this, has just been dismissed.

It is interesting to note the legal subtlety of the decision. Mr. Partridge is not dubbed "infamous" for advertising only, but rather for advertising after he said he would not, but the Master of the Rolls took occasion to very strongly condemn advertising by professional men in any case. The learned professions will evidently have to get on without advertising; and perhaps it is just as well.—*Printer's Ink.*

---

DEATH IN THE OPERATING CHAIR.—In *Zahntechnische Reform* is an account of the recent death of the opera singer, Frau Brandt-Geortz, of the Imperial theater in Hanover, which occurred under tragic circumstances in Cassell. Frau Brandt arrived there from Hanover April 11th, to visit her husband and family. She had complained for some time of violent pain in the right upper jaw caused apparently by a molar which was loose; there was also a fistulous opening on the gum. About midday she went to a dentist who was formerly well known to her, and who had frequently attended her, in order to have the tooth extracted. When Frau Brandt entered the room she was in such a very agitated condition, that as the dentist noticed also a marked increase of corpulence, he refused to undertake the extraction, especially as the patient wished for an anæsthetic. As she was, however, soon returning to Hanover, and wished to regain her health the dentist yielded. She took her seat in the chair but having complained of the disagreeable smell of the narcotic, requested the dentist to proceed with the extraction. This was successfully accomplished, but she must have suffered violent pain afterwards for she screamed loudly. The dentist succeeded in quieting her and handed her the usual glass of water to rinse out the mouth. She had hardly done so when the color of the face suddenly changed, and without uttering a sound sank back and died. Attempts to restore life by means of the electric current, ether injection and artificial respiration were kept up for three quarters of an hour but without avail. This would appear to be a case of syncope produced by mental emotion and the shock of extraction, with fatty degeneration of the heart. The patient was only 33 years of age.—*British Journal Dental Science.*

---

SINUS FROM AN ABSCESSED TOOTH.—By A. H. Beers, M. D., D. D. S.—On March 5th, 1892, an old man aged eighty years came to consult me about a "running sore" on the left side of his face. There was a mass of indurated tissue about the angle of the jaw, extending back to the mastoid process. There were two recently healed openings near the centre of this mass, with threatening renewal of suppuration.

History of case :—

Had never had toothache in his life. Last September his face began to swell. His wife, who is a know-it all creature, applied several linseed poultices, and proudly succeeded in bringing it to a "head" on his face. The old man had suffered great agony from neuralgic pains since last fall. He compared them to the repeated jabbing of a pen-knife all over the left side of his face and scalp. He had some trouble from trismus a short time ago, but it has entirely disappeared. His physician had lanced his face once. He then went to Montreal and consulted a homœopathic quack, who assured him it was cancer, gave him some pills, and told him he would soon be all right. I examined his face thoroughly, and came to the conclusion that it was a sinus from an abscessed tooth.

I examined his teeth, and found that he had a remarkably good set of teeth for his age. I found the putrid remains of the left upper dens-sapientia with a cheesy-looking exudate from around its neck. The lower dens-sapientia had a putrescent pulp, and was slightly tender on pressure. I removed both the upper and lower teeth and gave him a few simple directions. The upper I extracted as the cause of the sinus, and the lower one for several reasons. Its uselessness, the presence of putrescent pulp, incipient pericementitis, and the probable occurrence of alveolar abscess. Living as he did twenty miles from any doctor, he was not likely to consult any one until it had pointed and burst on his face. I endeavored to convince his wife of her error in poulticing the face for alveolar abscess, and, I hope, with success.

I have since heard (two weeks later) that the old man has had great relief from the neuralgic pains.—*Dominion Dental Journal.*

---

PROSECUTIONS.—Many licentiates have an idea that the members of the Board of Examiners should fulfil the duty of spies and detectives. If there is a quack in any obscure corner of any county they expect some officer of the Board to be omniscient as well as omnipresent, while they are particularly anxious to be *incognita* themselves. Nevertheless, though it is no more the duty of members of the Board than of others to secure information of illegal practice, it has been a duty voluntarily assumed, and most of the prosecutions have been brought about by the action of such officials. In Quebec

Province, especially, has this been the case, for the simple reason that if they had not been instigated by members of the Board they would have been altogether ignored. Last month two more cases were brought before the police court; one offender was fined $100 and costs, another $26 and costs. One of the most sublime exhibitions of cheek is attempted by the latter offender, who has been twice fined. Though he has been drawing a regular salary as an employee in a commercial firm, pretending to be indentured to a licentiate at the same time, and not having passed more than a few months in the office, he has applied to the local legislature for a private bill to permit him to practice without license, fee or examination. Quack premiers and boodling politicians reflect their principles upon every class of society, but there is hope at last for Quebec, as Mercier is degraded and honest men have hold of the helm.—*Dominion Dental Journal.*

---

HOW TO PROCURE AN IMPRESSION OF THE MOUTH WHEN PATIENT IS INCLINED TO NAUSEA AND VOMITING.——By C. V. Snelgrove, L. D. S., Toronto.—Get your druggist to make you some lozenges with one-quarter grain of cocaine in each lozenge. Before taking impression allow patient to dissolve one of these lozenges in mouth and swallow the spittle. If one is not sufficient, give patient another lozenge, allowing time enough for the lozenge to dissolve slowly, and you will find you can take an impression with plaster of paris without any inconvenience to patient or yourself.

Local anæsthetic for extraction of teeth or pulps:

| | | |
|---|---|---|
| R | Cocaine Hydrochlorate - - | grs. v. |
| | Acid carbolic xtals - - - | grs. iv. |
| | Gum Camphor opt - - - | grs. vi. |
| | Glycerine pure - - - | grs. xv. |
| | 95 per cent. spts vini Rect. Q. S. ad | ℥ ii |

M

Hypodermic syringe. Inject one or two drops deeply into the gums on inner and outer side of the tooth, and apply over the gums around the tooth, also in cavity of tooth, a piece of absorbent lint or cotton wet in the solution. Wait four or five minutes (by the watch) and the gums can be freely incised and tooth extracted with but little pain.—*Dominion Dental Journal.*

---

GOLD ALLOYS.—Prof. Roberts-Austen has drawn attention to the fact that the properties of gold are changed in a most remarkable manner by alloying it with small percentage of other metals, and he lately exhibited a new series of alloys of this metal with aluminum. One of these alloys, containing 20 per cent. of aluminum, forms an exception to the usual rule that the melting point of an alloy is lower than that of either of its constituents. This alloy has a fusing point above that of gold, the most infusible of its constituents. Curiously enough, the alloy with 10 per cent. of aluminum follows the ordinary rule. These alloys have the most brilliant colors. The 20 per cent. alloy is a brilliant ruby in tint, while those containing greater percentages of aluminum are purple in hue. —*Scientific American.*

---

A DIFFERENCE OF OPINION.—Dr. W. A. Johnston, M. D., D. D. S., says in *Review:* "The plan of heating a probe to dry out a pulp chamber is open to criticism. The probe to be of any service must be hot enough to have the same effect as a coagulating medicine and this we wish to avoid." He recommends Absolute Alcohol, and also air blasts, commencing with the cold air and increasing the temperature gradually, thus preventing coagulation by using hot air first. Dr. H. H. Silliman, another M. D., D. D. S., on the same subject and in the same journal says: "The Gilmer electric root dryer is the best thing I know for this purpose." He also says not to use alcohol because of its coagulating power. "Who shall decide?" when two such eminent men with equal number of degrees so radically antagonize each other.

